# Joint bayesian convolutional sparse coding for image super-resolution

**DOI:** 10.1371/journal.pone.0201463

**Published:** 2018-09-05

**Authors:** Qi Ge, Wenze Shao, Liqian Wang

**Affiliations:** 1 College of Telecommunications and Information Engineering, Nanjing University of Posts and Telecommunications, Nanjing, China; 2 National Engineering Research Center of Communications and Networking, Nanjing University of Posts and Telecommunications, Nanjing, China; Beijing University of Technology, CHINA

## Abstract

We propose a convolutional sparse coding (CSC) for super resolution (CSC-SR) algorithm with a joint Bayesian learning strategy. Due to the unknown parameters in solving CSC-SR, the performance of the algorithm depends on the choice of the parameter. To this end, a coupled Beta-Bernoulli process is employed to infer appropriate filters and sparse coding maps (SCM) for both low resolution (LR) image and high resolution (HR) image. The filters and the SCMs are learned in a joint inference. The experimental results validate the advantages of the proposed approach over the previous CSC-SR and other state-of-the-art SR methods.

## Introduction

Image super-resolution (SR) arms to reconstruct a high resolution (HR) image from a single or several low resolution (LR) image of the scene together [1–5]. The resolution limitations of low-cost imaging sensors is overcome by SR methods, and the degradation in the LR images caused by blur and the motion of camera or scene are utilized to reconstruct the HR image by SR methods. Since the motion parameters are estimated along with HR image solely from the LR images, SR is a difficult inverse problem, especially the single image SR.

To reconstruct a HR image from the LR image, information usually was lost during the down-sampling procedure, and more prior knowledge should be exploited. The errors are easily caused in estimating the missing pixels of HR image based on some simple assumptions that does not hold in many practical systems. For better reconstructing complicated structures in natural images, local priors are exploited in image patches by the single image super resolution (SISR) for estimating the HR image [6–9]. Example-based methods are one of the most important SISR methods. The existing example-based methods can be categorized into sparse coding-based and mapping-based methods [10–12]. Sparse coding-based methods train a couple of dictionaries for LR and HR image patches, and there are many approaches are proposed to build mapping functions between the LR and HR patches [13–16]. The mapping functions are learned using LR and HR patch pairs [6], [17–18]. For obtaining the final results, the pixels in the overlapped patches are averaged by the previous example-based methods. However, the pixels in the overlapped patches usually are not consistent, and the averaging process may destroy the inconsistency. Some approaches are proposed to overcome this problem and show the improved performance in image reconstruction [19].

Recently, the convolutional sparse coding based super-resolution (CSC-SR) method [19] utilizes a global sparse coding method for preserving the consistency better. CSC has been applied in unsupervised learning for visual features [20], [21]. It represents the input signal by the linear combination of N sparse feature maps corresponding to N filters. The number of filters in decomposing LR image is different from the number of filters in reconstructing HR image in CSC-SR method. By processing the global image rather than in each local patch, the previous CSC-SR has shown its outperformance over the sparse coding (SC) based ones. Although CSC-SR adopts a more adaptive decomposition-reconstruction strategy, this model still utilizes the fixed number of filters. Therefore, these parameters should be assigned a priori and a structure of latent space representing the input data should be applied to solve CSC problem. In that case, the sparse coding maps and the filters should be learned optimal. Otherwise, the errors in estimating the parameters may lead to instability in reconstructing the HR image.

To address the above problems, we present a novel convolutional sparse coding based super-resolution method. There are two stages in the proposed method. Firstly, we learn the filters and the sparse coding maps adaptively by modeling them using a Beta-Bernoulli distribution in decomposing the LR image. Secondly, we use the same distributions associated with the sparse coding maps when reconstructing the HR image. In each stage, the sparse coding maps are learned by minimizing the CSC problem with a variance Bayesian inference process. The Bernoulli distributions have an ability of controlling the frequency with the factors. Since learning processes on LR image and HR image are based on the same distribution, the filters and the sparse coding maps in each stage are inferred simultaneously under a joint inference process. The experimental results of the proposed method validate the competitive results with the state-of-the-art methods.

## Related works

Due to the consistency between the overlapped patches, the existing sparse coding-based methods [13–16] may smooth out the consistency including the high frequency edges and structures of the image. To preserve the consistency, a convolutional sparse coding is proposed to encode the whole image in [22]. The CSC based super-resolution (CSC-SR) method learns the sparse coding maps of the LR and HR image separately by solving the CSC problem. We use 3×3 low pass filter with all coefficients being 1/9 to extract the smooth component of the LR image, which is the same as the work [19]. Denote by **y** the LR image, by *Y*_*s*_ the smooth component of **y**, by *Y* the residual component of **y**. *Y* represents high frequency edges and texture structures. In CSC-SR, after the smooth component of LR image *Y*_*s*_ extracted by a low pass filter, and a group of LR filters are learned to decompose the residual component *Y*:

$$\min_{A,f}{\left\|Y-\sum_{i=1}^{N_{1}}f_{i}^{l}⊗A_{i}^{l}\right\|}_{F}^{2}+\lambda \sum_{i=1}^{N_{1}}{\|A_{i}^{l}\|}_{1},s.t.{\left\|f_{i}^{l}\right\|}_{F}^{2}\leq 1$$ (1)

where *Y* is the residual component including high frequency edges and texture structures, ${\left\{f_{i}^{l}\right\}}_{i=1\ldots N}$ are the filters, ${\left\{A_{i}^{l}\right\}}_{i=1\ldots N_{1}}$ are the sparse coding maps. ‖•‖_*F*_ represents the Frobenius norm and ‖•‖_1_ represents *l*-1 norm. ⊗ is convolution operator. And we use the initial sparse coding map provided on the website, http://www4.comp.polyu.edu.hk/~cslzhang/papers.htm. Gu et al. [19] use a stochastic average based alternating direction multiplier algorithm (SA-ADMM) to efficiently solve the CSC model (1) with a large number of filters. The HR image is also decomposed into a smooth component and residual component. *X* denotes the residual component which represents high frequency edges and structures of HR image. The *N*_1_-dimensional sparse coding space of $\left(A_{1}^{l},A_{2}^{l},\cdots A_{N_{1}}^{l}\right)$ can be transformed to *N*_2_-dimensional sparse coding space by multiply a trained mapping function $M\in R^{{}^{N_{1}\times N_{2}}}$ [19]. By amplifying the transformed matrix $\left(A_{1}^{l},A_{2}^{l},\cdots A_{N_{1}}^{l}\right)M$ with *k* factor, the column vectors of the transformed matrix are the initial the sparse coding map set ${\left\{A_{j}^{h}\right\}}_{j=1\ldots N_{2}}$. The filters and the mapping functions can be learned by solving another CSC minimization problem,

$$\min_{Z,f}{\left\|X-\sum_{j=1}^{N_{2}}f_{j}^{h}⊗A_{j}^{h}\right\|}_{F}^{2}+\lambda \sum_{j=1}^{N_{2}}{\left\|A_{j}^{h}\right\|}_{1},s.t.{\left\|f_{j}^{h}\right\|}_{F}^{2}\leq 1,m_{j}\underline{≻}0,{\left|m_{j}\right|}_{1}=1,$$ (2)

where $A_{j}^{h}=A^{l}\left(x,y\right)m_{j}$ represents the *j*-th sparse coding map of HR image, **M** represents the mapping function matrix of size *N*_1_×*N*_2_, ${\left\{f_{j}^{h}\right\}}_{j=1\ldots N_{2}}$ is the set of filters of HR image. After solving the minimization problem Eq (2) by SA-ADMM algorithm, the mapping function **M** and the filers $f_{j}^{h},j=1\ldots N_{2}$ can be learned and finally the residual component with high frequency texture structure of the HR image can be reconstruct by the summation of the convolution results of HR filters and the sparse coding maps $\hat{X}=\sum_{j=1}^{N_{2}}f_{j}^{h}⊗A_{j}^{h}$. By amplifying the smooth component of LR image *Y*_*s*_ with *k* factor, the smooth component of HR image *X*_*s*_ is obtained. The reconstructed HR image **x** is represented by $x=X_{s}+\hat{X}$.

## Joint bayesian convolutional sparse coding for image super-resolution

The performance of convolutional-sparse-coding (CSC)-based super resolution still depends on the appropriate choice of the unknown parameters [19]. To address the limitation, we present a joint Bayesian convolutional sparse coding framework for image super resolution (JB-CSC SR). Firstly, rather than the non-parameter Bayesian based sparse coding, we develop a new beta process to build the Bayesian based CSC model. Secondly, since we use different numbers of the filters and the corresponding sparse coding maps to decompose/reconstruct the LR and HR images, two CSC problems are solved by jointly learning a mapping function between the sparse coding maps in the two feature spaces.

### Bayesian based convolutional sparse coding model for decomposing LR image

In this paper, we propose a new CSC model for super resolution with Bayesian prior. As shown in S1 Fig, the SR model is a linear combination of N atoms with the corresponding coefficients. Likewise, the traditional CSC model is the linear combination of the convolutions with the corresponding linear coefficients, where all the linear coefficients equal to one. Therefore, adaptive linear coefficients should be added in the CSC model.

For conveniently describing our model, we transform the residual component of LR image ***Y***, the filters $f_{i=1\ldots N_{1}}^{l}$ and the sparse coding maps $A_{i}^{l}$ in Eq (1) into 1-dimensional, thus, we have the residual component *Y*∈*R*^*P*^, filters $f_{i=1\ldots N_{1}}^{l}\in R^{S}$, sparse coding maps $a_{i=1\ldots N_{1}}^{l}\in R^{\left(m+s-1\right)\left(n+s-1\right)}$, where *P* = *m*×*n*, *S* = *s*×*s*. The number of filters for decomposing the LR image is *N*_1_. $d_{i}^{l}\in R^{P}$ is the *i-*th 1-dimensional version of the convolution $d_{i}^{l}=f_{i}^{l}⊗a_{i}^{l}$. With the base measure ℏ_0_ and the parameters *a*,*b*>0, a representation of Beta Process is as follows,

$$\hbar =\sum_{i=1}^{N_{1}}\pi_{i}\delta_{d_{i}^{l}},\;\pi_{i}∼\mathrm{Beta}\left(a/N_{1},b\left(N_{1}-1\right)/N_{1}\right)$$ (3)

where $\delta_{d_{i}^{l}}$ is unit point mass at $d_{i}^{l}$. A draw ℏ from the process is a set of *N* probabilities $\pi_{i=1\ldots N_{1}}$, each associated with $d_{i=1\ldots N_{1}}^{l}$ that are drawn *i*.*i*.*d*. from the base measure ℏ_0_. Considering *π*_*i*_ to be a Bernoulli distribution parameter, we use ℏ to draw a binary vector $z^{l}=\left(z_{1}^{l},z_{2}^{l},\cdots ,z_{N_{1}}^{l}\right)\in {\mathbb{R}}^{N_{1}}$, $z_{i}^{l}\in {\left\{0,1\right\}}^{N_{1}}$, $z^{l}∼\prod_{i=1}^{N_{1}}\mathrm{Bernoulli}\left(\pi_{i}\right)$. In the limit *N*_1_→∞, the number of the non-zero elements in **z**^*l*^ itself is derived from Poisson(*a*/*b*) that control the number of used convolutions $d_{i}^{l},i=1\ldots N_{1}$. Concatenating the convolutions $d_{i}^{l},i=1\ldots N_{1}$ together, we have the convolution matrix $D^{l}=\left(d_{1}^{l},d_{2}^{l},\cdots ,d_{N_{1}}^{l}\right)$. Since the residual component *Y* can be represented as the linear combination of the convolutions $d_{1}^{l},d_{2}^{l},\cdots ,d_{N_{1}}^{l}$, it can be expressed in the following term,

$$Y=D^{l}w^{l}+\varepsilon^{l}$$ (4)

where $w^{l}={\left(w_{1}^{l},w_{2}^{l},\cdots ,w_{N_{1}}^{l}\right)}^{T}\in {\mathbb{R}}^{N_{1}}$ is the coefficient vector for linearly representing **y**. *ε*^*l*^∈ℝ^*P*^ denotes the error term, $D^{l}=\left(d_{1}^{l},d_{2}^{l},\cdots ,d_{N_{1}}^{l}\right)=\left(f_{1}^{l}⊗a_{1}^{l},f_{2}^{l}⊗a_{2}^{l},\cdots f_{N_{1}}^{l}⊗a_{N_{1}}^{l}\right)$. With the binary vector $z^{l}\in {\mathbb{R}}^{N_{1}}$, the parameters in Eq (5) can be expressed as,

$$w^{l}=z^{l}⊙s^{l},s^{l}∼N\left(0,\gamma_{s}{}^{-1}I_{N_{1}}\right),\varepsilon^{l}∼N\left(0,\gamma_{\varepsilon }{}^{-1}I_{P}\right),$$

$$f_{i}^{l}∼N\left(0,S^{-1}I_{S}\right)d_{i}^{l}∼N\left(0,P^{-1}I_{P}\right)$$ (5)

where ⊙ represents the Hadamard vector product, $I_{N_{1}}$, **I**_*S*_, and **I**_*P*_ represent the identity matrix with size of *N*_1_×*N*_1_, *S*×*S*, *P*×*P*, respectively. *γ*_*s*_~Gamma(*c*,*d*), *γ*_*ε*_~Gamma(*e*,*f*) are drawn from the Gamma distributions, respectively. Following the model Eq (5) and the general structure of beta process described in [23], the convolutional sparse coding model with Bayesian prior can be expressed as,

$$\min_{a,f}{\left\|Y-\left(f_{1}^{l}⊗a_{1}^{l},f_{2}^{l}⊗a_{2}^{l},\cdots f_{N_{1}}^{l}⊗a_{N_{1}}^{l}\right)w^{l}\right\|}_{F}^{2}+\lambda \sum_{i=1}^{N_{1}}{\left\|a_{i}^{l}\right\|}_{1},s.t.{\left\|f_{i}^{l}\right\|}_{F}^{2}\leq 1$$ (6)

where **w**^*l*^ is defined as in Eq (5). We rewrite the formula Eq (6) as follows,

$$\min_{a,f}{\left\|Y-\sum_{i=1}^{N_{1}}f_{i}^{l}⊗a_{i}^{l}w_{i}^{l}\right\|}_{F}^{2}+\lambda \sum_{i=1}^{N_{1}}{\left\|a_{i}^{l}w_{i}^{l}\right\|}_{1},s.t.{\left\|f_{i}^{l}\right\|}_{F}^{2}\leq 1$$ (7)

Eq (7) is a typical convolutional sparse coding minimization problem with Bayesian priors. The weighted linear combination of the convolutions, $\sum_{i=1}^{N_{1}}f_{i}^{l}⊗a_{i}^{l}w_{i}^{l}$, is equivalent to the summation of the convolution of the filters $f_{i}^{l},i=1\cdots N_{1}$ and the weighted sparse coding maps $a_{i}^{l}w_{i}^{l}$, *i* = 1⋯*N*_1_. Analogous to [19], we adopt the SA-ADMM algorithm to solve the minimization problem Eq (7) to train the filters $f_{i=1\ldots N_{1}}^{l}$ and learn the new sparse coding maps $a_{i}^{l}w_{i}^{l},i=1\ldots N_{1}$ with the Bayesian prior.

### Bayesian based convolutional sparse coding model for reconstructing HR image

Authors in [19] train the mapping function to obtain the sparse coding maps of the HR image from the sparse coding maps of the LR image. However, inappropriate choosing the unknown parameters, including the number of the convolutions for HR image and the parameters of the mapping function, may lead to instability of the performance of the algorithm. To address this problem, we build the convolutional sparse coding (CSC) model with Bayesian prior to learn the HR filters to reconstruct the HR image. For convenient expression, given the sparse coding maps learned from Eq (7), we have $A^{l}=\left(a_{1}^{l}w_{1}^{l},\ldots ,a_{N_{1}}^{l}w_{N_{1}}^{l}\right)\in {\mathbb{R}}^{\left(m+s-1\right)\left(n+s-1\right)\times N_{1}}$. *N*_2_ is the number of filters for reconstructing the HR image. The authors in [19] transform **A**^*l*^ from *N*_1_-dimension feature space to the *N*_2_-dimension feature space by right-multiplying it with the mapping function $M\in {\mathbb{R}}^{{}^{N_{1}\times N_{2}}}$, ($m_{j}\underline{≻}0,{\left|m_{j}\right|}_{1}=1$, **m**_*j*_ is the *j*-th column of **M**), they have $A^{l}M\in {\mathbb{R}}^{\left(m+s-1\right)\left(n+s-1\right)\times N_{2}}$. And then by zooming each column of **A**^*l*^**M** with *k* factor, the sparse coding maps $A^{h}={\left(a_{j}^{h}\right)}_{j=1\ldots N_{2}}\in {\mathbb{R}}^{k\left(m+s-1\right)\left(n+s-1\right)\times N_{2}}$ for HR image is obtained. Different from [19], we apply Bayesian prior to the mapping function **M**, the HR filters, and the sparse coding maps together.

Let *k* denote the zooming factor, thus we represent the 1-dimensional version of the residual component of HR image representing high frequency edges and structures as *X*∈ℝ^*kP*^. We represent the mapping function as $M=\left(m_{1},m_{2},\cdots ,m_{N_{2}}\right)\in {\mathbb{R}}^{{}^{N_{1}\times N_{2}}}$, the HR filters as $f_{j}^{h}\in {\mathbb{R}}^{kS},j=1\ldots N_{2}$, and the sparse coding maps as $a_{j=1\ldots N_{2}}^{h}\in {\mathbb{R}}^{k\left(m+s-1\right)\left(n+s-1\right)}$. Let $d_{j}^{h}\in {\mathbb{R}}^{kP}$ denote the *j-*th 1-dimensional version of the convolution $d_{j}^{h}=f_{j}^{h}⊗a_{j}^{h}$. We initialize each column of mapping function **M** as $m_{j}∼N\left(0,N_{1}{}^{-1}I_{N_{1}}\right)$. With the same base measure ℏ_0_ and the same parameters *a*,*b*>0 in Eq (3), we have a representation of Beta Process as follows

$$\hbar^{h}=\sum_{j=1}^{N_{2}}\pi_{j}\delta_{d_{j}^{h}},\;\pi_{i}∼\mathrm{Beta}\left(a/N_{2},b\left(N_{2}-1\right)/N_{2}\right)$$ (8)

where $\delta \left(d_{j}^{h}\right)$ is unit point mass at $d_{j}^{h}$. Similar to the Beta Process in Sub-section Ⅲ-A, we also have ℏ to draw a binary vector $z^{h}=\left(z_{1}^{h},z_{2}^{h},\cdots ,z_{N_{2}}^{h}\right)\in {\mathbb{R}}^{N_{2}}$, $z_{j}^{h}\in {\left\{0,1\right\}}^{N_{2}}$, $z^{h}∼\prod_{j=1}^{N_{2}}\mathrm{Bernoulli}\left(\pi_{j}\right)$. The number of the non-zero elements in **z**^*h*^ itself is drawn from Poisson(*a*/*b*) and it controls the number of used convolutions $d_{j}^{h},j=1\ldots N_{2}$, when *N*_2_→∞. Given the binary vector **z**^*h*^, we have the diagonal matrix $\Lambda =diag\left(z^{h}\right)\in {\mathbb{R}}^{N_{2}\times N_{2}}$. By multiplying the mapping function **M** with Λ, we have $M\Lambda =\left(z_{1}^{h}m_{1},z_{2}^{h}m_{2},\cdots ,z_{N_{2}}^{h}m_{N_{2}}\right)$, thus the columns of the mapping function is weighted by the elements of **z**^*h*^. We represent the sparse coding maps as $A_{\Lambda }^{h}={\left(a_{\Lambda j}^{h}\right)}_{j=1\ldots N_{2}}$ for HR image. First, we transform **A**^*l*^ with *N*_1_ sparse coding maps for LR image to **A**^*l*^**M**Λ with *N*_2_ sparse coding maps by the new mapping function **M**Λ. By zooming **A**^*l*^**M**Λ with *k* factor, we have the sparse coding maps $A_{\Lambda }^{h}\left(x,:\right)=A^{l}M\Lambda \left(x/k,:\right)$, where x represents the horizontal coordinate in the image domain.

Given the convolution matrix $D^{h}=\left(d_{1}^{h},d_{2}^{h},\cdots ,d_{N_{2}}^{h}\right)$, the residual component **x** of HR image can be represented as a linear combination of the convolutions, $d_{1}^{h},d_{2}^{h},\cdots ,d_{N_{2}}^{h}$,

$$X=D^{h}w^{h}+\varepsilon^{h},$$ (9)

where $w^{h}={\left(w_{1}^{h},w_{2}^{h},\cdots ,w_{N_{2}}^{h}\right)}^{T}\in {\mathbb{R}}^{N_{2}}$ and **ε**^*h*^∈ℝ^*kP*^ represent the coefficient vector and the error term, respectively. The coefficient vector **w**^*h*^ can be decomposed by the binary vector $z^{h}\in {\mathbb{R}}^{N_{2}}$. Similar to Eq (5), we have,

$$w^{h}=z^{h}⊙s^{h},s^{h}∼N\left(0,\gamma_{s}{}^{-1}I_{N_{2}}\right),\varepsilon^{h}∼N\left(0,\gamma_{\varepsilon }{}^{-1}I_{kP}\right),$$

$$f_{j}^{h}∼N\left(0,kS^{-1}I_{kS}\right),\;d_{j}^{h}∼N\left(0,kP^{-1}I_{kP}\right)$$ (10)

$I_{N_{2}}$, **I**_*kS*_, **I**_*kP*_ represent the identity matrix with size of *N*_2_×*N*_2_, *kS*×*kS*, *kP*×*kP*, respectively. *γ*_*s*_ and *γ*_*ε*_ are the defined as in Eq (5). Following the parameter settings in Eq (10), the convolutional sparse coding model with Bayesian prior for learning the HR filters can be expressed as,

$${\left\{f^{h},a_{\Lambda }^{h}\right\}}_{j}=\min_{a,f}{\left\|X-\left(f_{1}^{h}⊗a_{\Lambda 1}^{h},f_{2}^{h}⊗a_{\Lambda 2}^{h},\cdots f_{N_{2}}^{h}⊗a_{\Lambda N_{2}}^{h}\right)w^{h}\right\|}_{F}^{2}+\lambda \sum_{j=1}^{N_{2}}{\left\|a_{\Lambda j}^{h}\right\|}_{1},s.t.{\left\|f_{j}^{h}\right\|}_{F}^{2}\leq 1$$ (11)

where **w**^*h*^ is defined as in Eq (10). $\left(f_{1}^{h}⊗a_{\Lambda 1}^{h},f_{2}^{h}⊗a_{\Lambda 2}^{h},\cdots f_{N_{2}}^{h}⊗a_{\Lambda N_{2}}^{h}\right)w^{h}$ represents the weighted linear combination of the convolutions, we rewrite the formula Eq (11) as follows,

$$\left\{f^{h},a_{\Lambda }^{h}\right\}=\min_{a,f}{\left\|X-\sum_{j=1}^{N_{2}}f_{j}^{h}⊗a_{\Lambda j}^{h}w_{j}^{h}\right\|}_{F}^{2}+\lambda \sum_{j=1}^{N_{2}}{\left\|a_{\Lambda j}^{h}w_{j}^{h}\right\|}_{1},s.t.{\left\|f_{j}^{h}\right\|}_{F}^{2}\leq 1$$ (12)

Eq (12) is also a convolutional sparse coding minimization problem with Bayesian priors. We solve it by SA-ADMM algorithm. After training the HR filters ${\left\{f_{j}^{h}\right\}}_{j}$, the sparse coding maps ${\left\{a_{\Lambda j}^{h}\right\}}_{j}$, and the mapping function **M**Λ, the residual component of HR image is formulated as the summation of the convolutions of HR filters and the sparse coding maps: $\hat{X}=\sum_{j=1}^{N_{2}}f_{j}^{h}⊗a_{\Lambda j}^{h}w_{j}^{h}$. The estimation of HR image is reconstructed by combing the residual component $\hat{X}$ and the smooth component. The training framework of the proposed JB-CSC super-resolution method is shown in S2 Fig.

### Variance bayesian inference process

Gibbs sampling is usually used to perform Bayesian inference to sample the parameters from the conditional distribution of each hidden variable, given the other observations [24]. The authors in [25] update the parameters according to the posterior distributions over the model for Gibbs sampler. The inference process performs updating. It is also called sampling over these posteriors. Inspired by [23], [25], we derive analytical expressions for the Gibbs sample, starting with the convolutions ${\left\{d_{i}^{l}\right\}}_{i=1\ldots N_{1}}$ of the LR image instead of the dictionary in their models [25]. For our model, we derive the following about the posterior distribution over a LR convolution to sampling the *i*-th convolution,

$$p\left(d_{i}^{l}|-\right)\propto \prod_{i=1}^{N_{{}_{1}}}N\left(y|D^{l}\left(z^{l}⊙s^{l}\right),P^{-1}I_{P}\right)$$ (13)

Let $y_{d_{i}^{l}}$ denote the contribution of the convolution $d_{i}^{l}$ to the residual component of LR image **y**, then we have,

$$y_{d_{i}^{l}}=y-D\left(z^{l}⊙s^{l}\right)+d_{i}^{l}\left(z^{l}⊙s^{l}\right)$$ (14)

With $y_{d_{i}^{l}}$, the posterior distribution in Eq (14) can be rewritten as,

$$p\left(d_{i}^{l}|-\right)\propto \prod_{i=1}^{N_{{}_{1}}}N\left(y_{d_{i}^{l}}|D^{l}\left(z^{l}⊙s^{l}\right),\gamma_{\varepsilon }{}^{-1}I_{P}\right)N\left(d_{i}^{l}|0,P^{-1}I_{P}\right)$$ (15)

Given Eq (15), the posterior distribution over a convolution can be written as

$$p\left(d_{i}^{l}|-\right)\propto N\left(d_{i}^{l}|\mu_{i},P^{-1}I_{P}\right)$$ (16)

where $\mu_{i}=\frac{\gamma_{\varepsilon }}{S_{i}}\sum_{i=1}^{N_{1}}\left(z_{i}^{l}⊙s_{i}\right)y_{d_{i}^{l}}$, $P_{i}=P+\gamma_{\varepsilon }{\left(z_{i}^{l}⊙s_{i}\right)}^{2}$.

Once the convolutions have sampled, we sample the parameter $z_{i}^{l}$. By the distribution of the *i*-th convolution, the posterior probability distribution over $z_{i}^{l}$ can be expressed as

$$p\left(z_{i}^{l}|-\right)\propto N\left(y_{d_{i}^{l}}|d_{i}^{l}\left(z_{i}^{l}⊙s_{i}^{c}\right),\gamma_{\varepsilon }{}^{-1}I_{P}\right)\mathrm{Bernoulli}\left(z_{i}^{l}|\pi_{i}\right).$$ (17)

With the prior probability of $z_{i}^{l}=1$ given by *π*_*i*_, we can write the following posterior probability,

$$p\left(z_{i}^{l}=1|-\right)\propto \pi_{i}\exp\left(-\frac{\gamma_{\varepsilon }}{2}{\left\|y_{d_{i}^{l}}-d_{i}^{l}s_{i}\right\|}_{2}^{2}\right).$$ (18)

The prior probability of $z_{i}^{l}=0$ is given by 1−*π*_*i*_, the posterior probability can be derived as,

$$p\left(z_{i}^{l}=0|-\right)\propto {\left(1-\pi \right)}_{i}\exp\left(-\frac{\gamma_{\varepsilon }}{2}{\left\|y_{d_{i}^{l}}\right\|}_{2}^{2}\right)$$ (19)

Analogous to the K-SVD algorithm, we derive the expression of sampling $z_{i}^{l}$,

$$z_{i}^{l}∼\mathrm{Bernoulli}\left(z_{i}^{l}|\frac{\pi_{i}\zeta }{1+\pi_{i}\left(\zeta -1\right)}\right),\;\zeta =\exp\left(-\frac{\gamma_{\varepsilon }s_{i}}{2}\left({\left\|d_{i}^{l}\right\|}_{2}^{2}s_{i}-2{\left(y_{d_{i}^{l}}\right)}^{T}d_{i}^{l}\right)\right)$$ (20)

Having sampled the convolution and the binary vector, we adopt the method of sampling of **s**^*l*^, *γ*_*s*_, *π*_*i*_, *γ*_*ε*_, according to [25].

### Summary of algorithm

The proposed model consists of two stages: first, we solve the CSC model problem to learn LR filters and the sparse coding maps; second, we solve the CSC problem to learn the HR filters, the sparse coding maps, and the mapping function for reconstructing the HR image. By incorporating the Bayesian prior and the inference process into the convolutional sparse coding model, we summarize the algorithm of the proposed method in **Algorithm 1**.

### Algorithm 1

1. Input the train LR image *Y*, decompose it into smooth component *Y*_*s*_ and residual component **y**;

2. Initialize the parameters **z**^*l*^, **s**^*l*^, **ε**^*l*^, $d_{i}^{l}$, and $f_{i=1\ldots N_{1}}^{l}$ as in Eq (5).

2. Solving Eq (6) by SA-ADMM algorithm to obtain $f_{i=1\ldots N_{1}}^{l}$ and the weighted sparse coding maps $A^{l}={\left(a_{i}^{l}w_{i}^{l}\right)}_{i=1\ldots N_{1}}$ while updating the parameters for **z**^*l*^ by Eq (20), and updating **s**^*l*^,**ε**^*l*^, $f_{i=1\ldots N_{1}}^{l}$ accordingly. Then the smooth component *Y*_*s*_ is zoomed into *X*_*s*_ with *k* factor.

3. Initialize $M=\left(m_{1},m_{2},\cdots ,m_{N_{2}}\right)\in {\mathbb{R}}^{{}^{N_{1}\times N_{2}}}$, $m_{j}∼N\left(0,S^{-1}I_{N_{1}}\right)$ and the parameters **z**^*h*^, **s**^*h*^, **ε**^*h*^, $d_{j}^{h}$, and $f_{j=1\ldots N_{2}}^{h}$ as in Eq (10);

4. Transform the sparse coding maps $A^{l}={\left(a_{i}^{l}w_{i}^{l}\right)}_{i=1\ldots N_{1}}$ to **A**^*l*^**M**Λ, where $\Lambda =diag\left(z^{h}\right)\in {\mathbb{R}}^{N_{2}\times N_{2}}$, by zooming it with *k* factor, get the initial sparse coding maps $A_{\Lambda }^{h}$ for HR image;

5. Solving Eq (12) by SA-ADMM algorithm to obtain the filters $f_{j=1\ldots N_{2}}^{h}$, sparse coding maps $A_{\Lambda }^{h}$, and the mapping function **M** while updating the parameters for **z**^*h*^ analogous to Eq (20), and updating **s**^*h*^, **ε**^*h*^ accordingly.

6. Reconstruct the residual component ***X*** of HR image by $\hat{x}=\sum_{j=1}^{N_{2}}f_{j}^{h}⊗a_{\Lambda j}^{h}w_{j}^{h}$. The final HR image is obtained by $X=X_{s}+\hat{x}$

## Experimental results

In this section, we compare the proposed joint Bayesian convolutional sparse coding (JB-CSC) method with four state-of-the-art SR methods, including the Beta process joint dictionary learning method (BPJDL) [16], convolutional neural network based method (CNN-SR) [18], convolutional sparse coding based method (CSC-SR) [19], and adjusted ANR (A-ANR) [26]. We use the source code provided in the authors' website, and use the parameters which they recommend. The original ground truth images are downsized by bi-cubic interpolation to generate LR-HR image pairs for both training and evaluation.

### Parameter setting

To have a fair comparison between CSC-SR [19] and our model, we adopt the same training set provided in [19], the same regularization parameters, and the same implementation on the image boundary. The beta-distribution parameters are set as *a* = *N*_1_ for LR image and *a* = *N*_2_ for HR image, and b = 1 for both HR and LR image. The hyper-parameters in Eq (5) and Eq (10) were set as *c* = *d* = *e* = *f* = 10^−6^, which is provided in [24]. All these parameters in the Bayesian inference process have not been tuned. We conduct all the comparison experiments on a PC with Intel Core i7 25.GHz CPU 4GB RAM on Matlab platform.

### Comparison with state-of-the-arts

We compare the proposed JB-CSC method with other recent SR methods on the widely used test images from Set5 [27] and Set14 [28] for different zooming factors. The test images of Set5 and Set14 are available at https://sites.google.com/site/jbhuang0604/publications/struct_sr. These two test image datasets are both publicly released and popularly usedfor image super-resolution works [1–6], [8–26] without any consent or any copyright. We show the SR results of BPJDL [16], A-ANR [26], CNN-SR [18], convolutional sparse coding based method (CSC-SR) [19], and the proposed JB-CSC method in S3–S6 Figs. In S3 Fig, we show the SR results of image *Bird* with zooming factor *k* = 2. In S4, S5 and S6 Figs we show the SR results of image *Foreman*, *Face Butterfly* with zooming factor *k* = 3. And the PSNR values by the competing methods are shown in Table 1.

**Table 1:** PSNR results of different methods.

	Zooming factor *k* = 2					Zooming factor *k* = 3				
	BPJDL	CNN	A-ANR	CSC	JB-CSC	BPJDL	CNN	A-ANR	CSC	JB-CSC
Butterfly	31.43	32.20	31.94	31.96	**32.02**	26.42	27.58	27.22	27.11	**27.60**
Face	35.75	35.60	35.72	35.71	**35.76**	33.45	33.57	33.74	33.80	**33.94**
Bird	40.99	40.63	40.98	41.49	**41.64**	34.53	34.92	34.48	35.78	**35.90**
Woman	35.23	34.93	35.27	35.31	**35.37**	30.50	30.92	31.19	31.27	**31.42**
Foreman	36.49	36.19	**36.91**	36.64	36.79	32.91	33.34	34.22	34.24	**34.33**
Coast	30.60	30.49	30.55	30.65	**30.71**	27.07	27.19	27.27	27.27	**27.38**
Flowers	32.91	33.04	33.03	33.15	**33.22**	28.62	28.98	29.05	29.05	**29.15**
Zebra	33.62	33.30	33.67	33.77	**33.83**	28.73	28.90	29.06	29.30	**29.43**
Lena	36.58	36.48	36.57	36.66	**36.76**	33.13	33.39	33.50	33.62	**33.75**
Bridge	27.77	27.70	27.78	27.84	**27.91**	24.99	25.07	25.17	25.20	**25.33**
Baby	38.54	38.41	38.43	38.48	**38.53**	35.15	35.00	35.14	35.28	**35.44**
Peppers	36.71	36.75	**37.06**	36.90	36.97	34.02	34.35	34.71	34.72	**34.86**
Man	30.80	30.82	30.88	30.97	**31.02**	28.05	28.18	28.29	28.34	**28.44**
Barbara	28.68	28.59	28.70	28.77	**28.81**	26.82	26.65	26.47	26.67	**26.78**
Ave	34.01	33.94	34.11	34.16	**34.24**	30.31	30.57	30.68	30.83	**30.97**

In S3 Fig, we can see from the small window in the right-bottom of the images, the SR results by CNN-SR over-smooth the edge. And very little ringing artifacts are generated by BPJDL, A-ANR, and CSC, since the zooming factor is low. The PSNR values of the results at this factor *k* = 2 show the outperformance of our method.

In S4 Fig, in the small window of the images, the SR results of the competing methods, such as BPJDL and A-ANR show that more edges are over-smoothed. Comparing CSC-SR and the proposed method, the edges of the image can be preserved better than CSC-SR.

In S5 Fig, the results of the proposed method are compared with the results of BPJDL, A-ANR, CNN-SR, and CSC-SR. In the small windows, we can see that more textures of the hair are preserved much better in BPJDL. This comparison verifies that the models with Bayesian prior, such as BPJDL and the proposed method, have the outperformance in preserving the texture details of the images.

In S6 Fig, the proposed method is compared with the competing methods with the zooming factor *k* = 4. In (b), we can see that the result of BPJDL shows that the ringing artifacts increase as the zooming factor increases. As shown in (d), (c), (e), (f) of S6 Fig, the ringing artifacts remain in the results of A-ANR, CSC-SR and the proposed method while not in the result of CNN-SR. However, the edges are over-smoothed by CNN-SR. The PSNR value of the proposed method is higher than the competing methods.

## Conclusion

In this paper, we present a convolutional sparse coding based super resolution method with a joint Bayesian learning strategy (JB-CSC). JB-CSC employs a coupled Beta-Bernoulli process to incorporate the Bayesian prior into the convolutional sparse coding model, which avoids instability caused by estimating the unknown parameter. Different from the CSC method, the filters and sparse feature maps for both low resolution (LR) image and high resolution (HR) are learned adaptively through the Bayesian learning strategy. The experimental results validate the advantages of the proposed approach, compared with the previous CSC-SR and other state-of-the-art SR methods.

## Supporting information

S1 Fig The difference between the sparse coding model and the convolutional sparse coding model. (TIF)

S2 Fig Pipeline of the proposed JB-CSC super-resolution training framework. (TIF)

S3 Fig Super resolution results on image *Bird* by different algorithms. (TIF)

S4 Fig Super resolution results on image *Foreman* by different algorithms. (TIF)

S5 Fig Super resolution results on image *Face* by different algorithms. (TIF)

S6 Fig Super resolution results on image *Butterfly* by different algorithms. (TIF)

S1 Dataset (ZIP)
