# Supplementary figures and images for: Joint bayesian convolutional sparse coding for image super-resolution
Source: PLoS One. 2018 Sep 5;13(9):e0201463. doi: 10.1371/journal.pone.0201463 (PMC6124716; doi:10.1371/journal.pone.0201463)

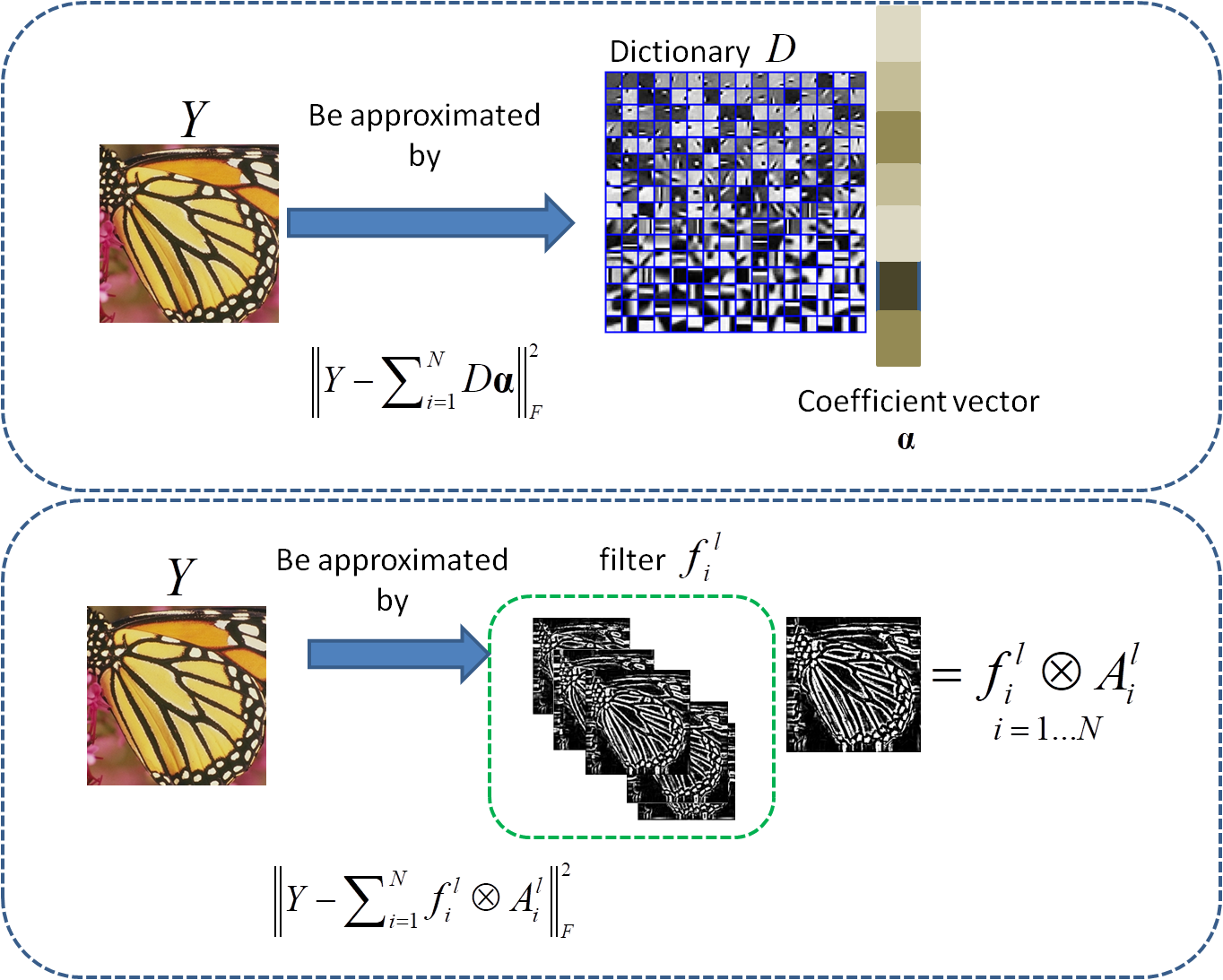

Supplement: S1 Fig — (TIF) [file pone.0201463.s001.tif]

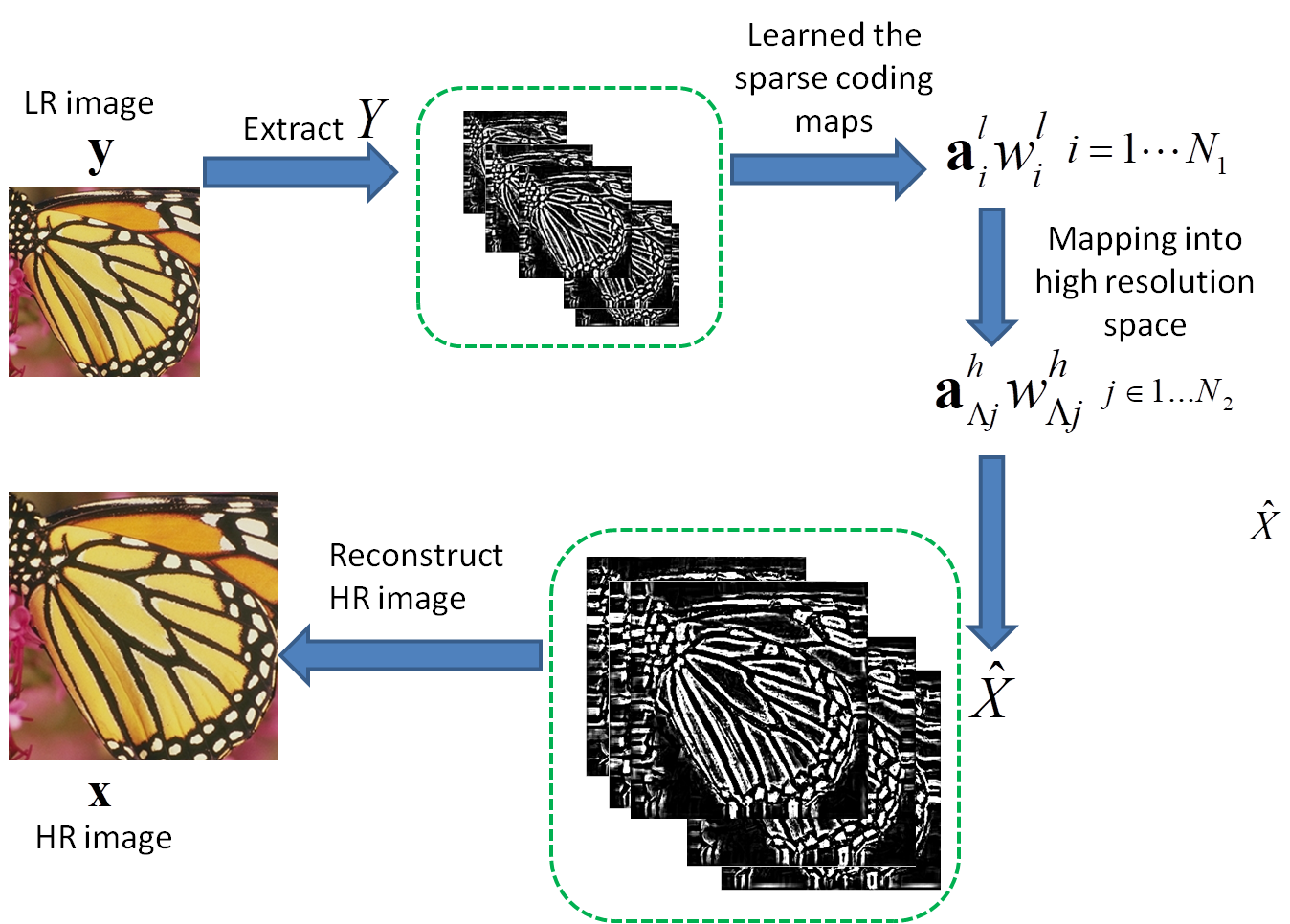

Supplement: S2 Fig — (TIF) [file pone.0201463.s002.tif]

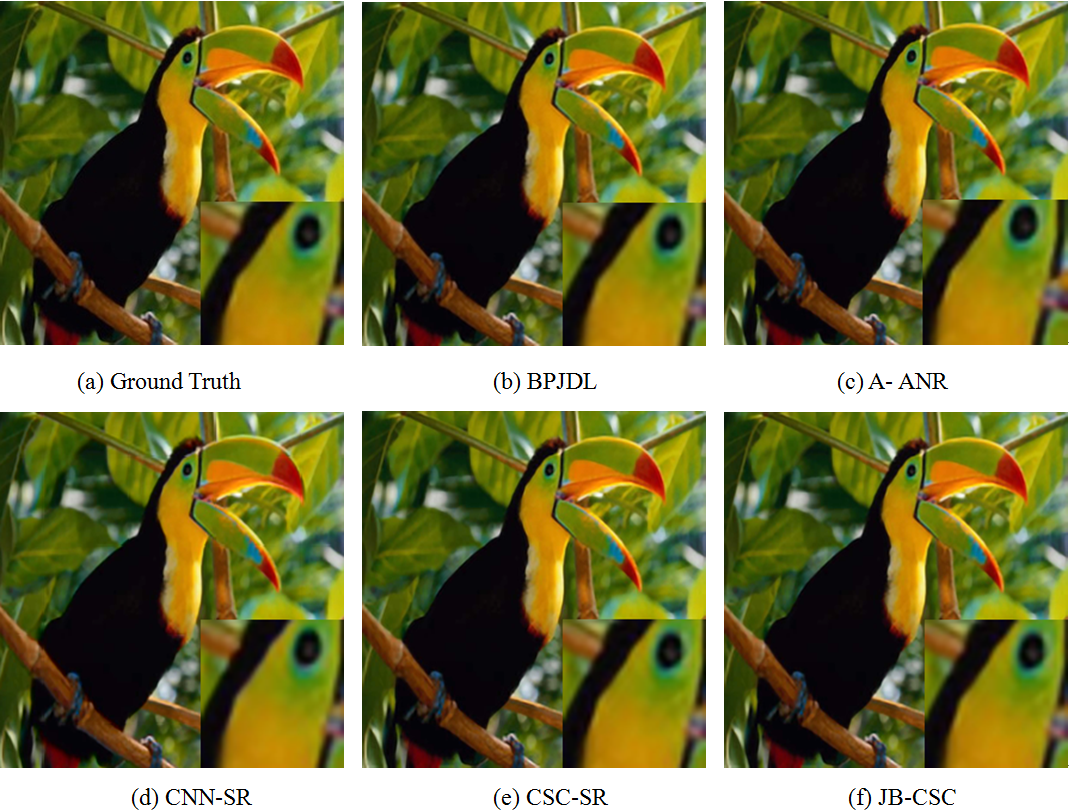

Supplement: S3 Fig — (TIF) [file pone.0201463.s003.tif]

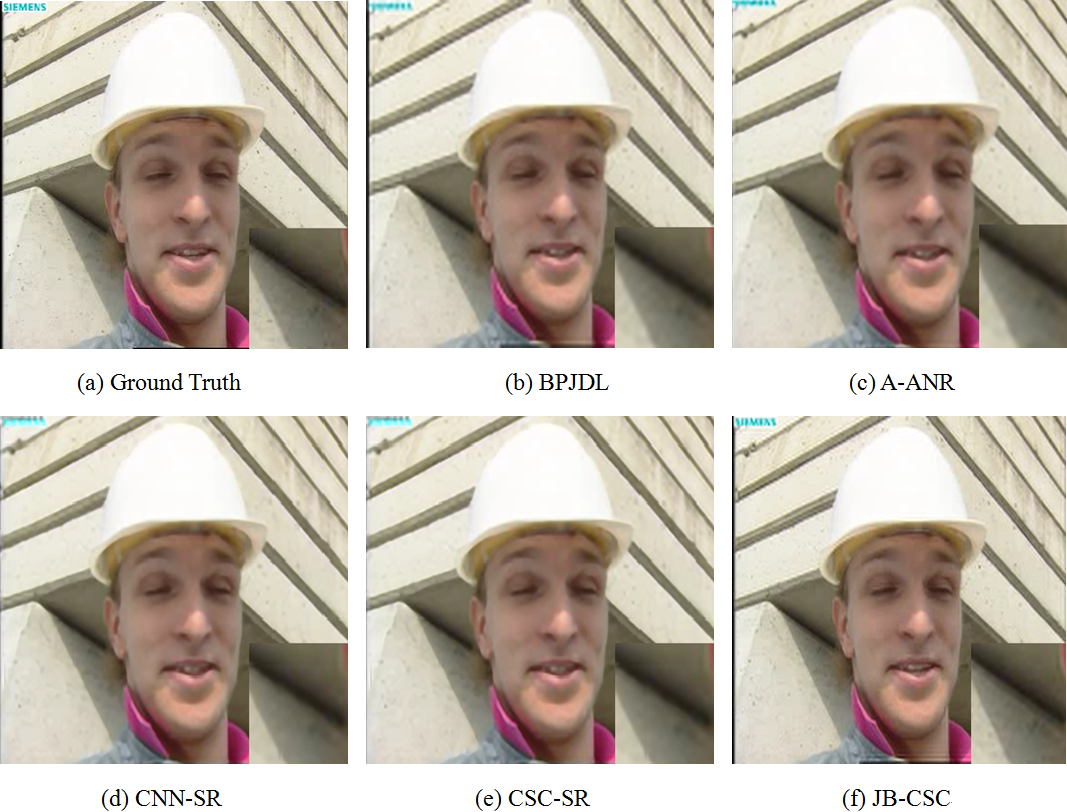

Supplement: S4 Fig — (TIF) [file pone.0201463.s004.tif]

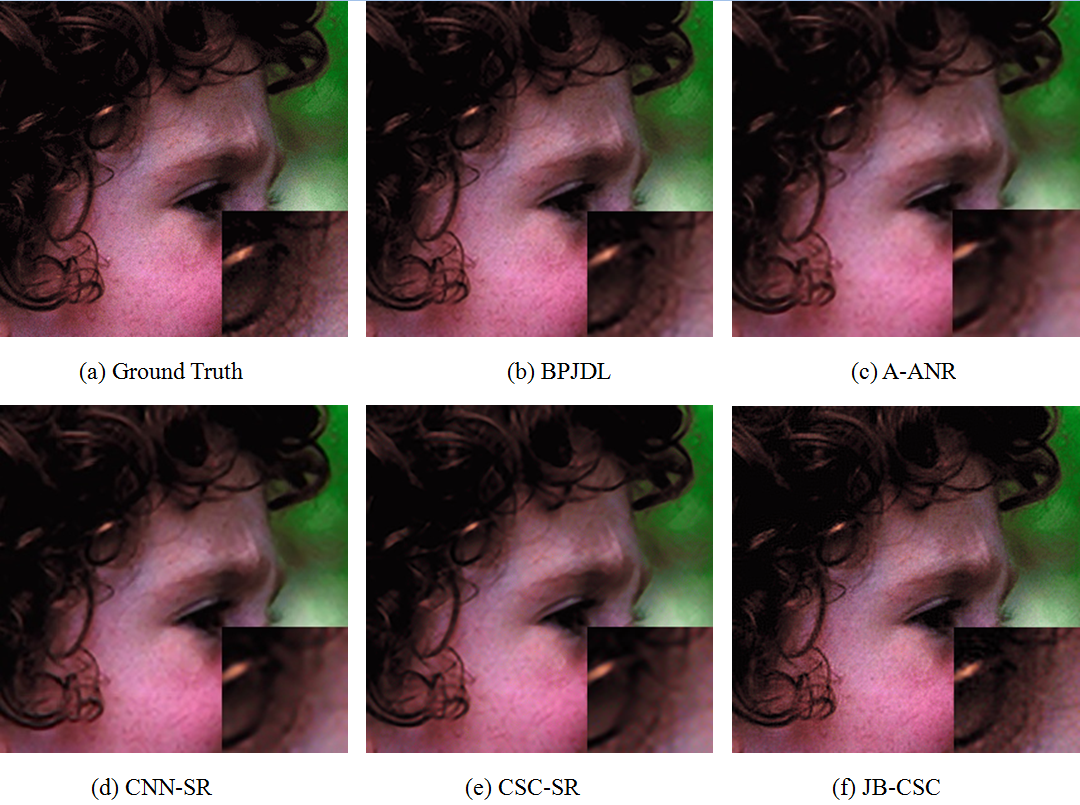

Supplement: S5 Fig — (TIF) [file pone.0201463.s005.tif]

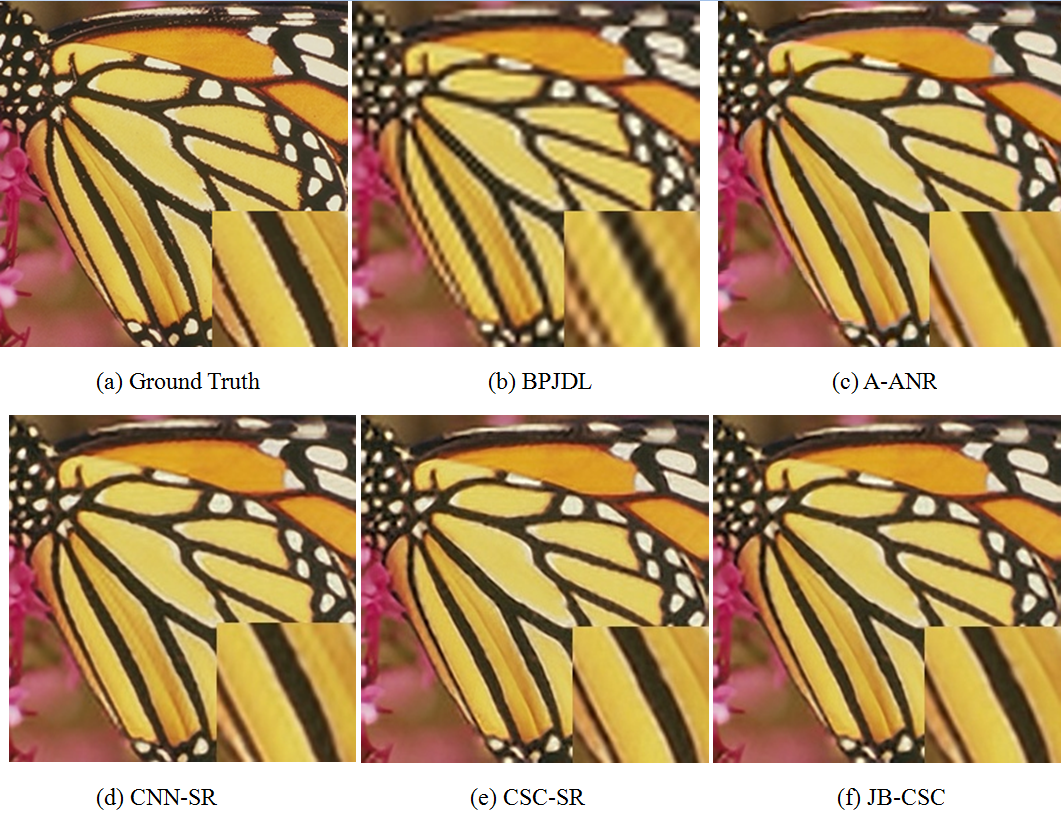

Supplement: S6 Fig — (TIF) [file pone.0201463.s006.tif]

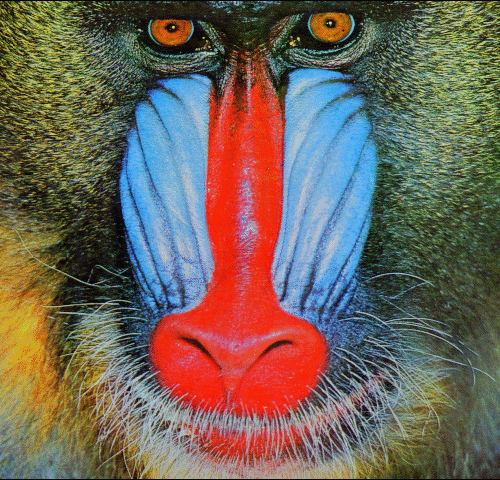

Supplement: S1 Dataset — (ZIP) [file pone.0201463.s007.zip › data/Set14/baboon.bmp]

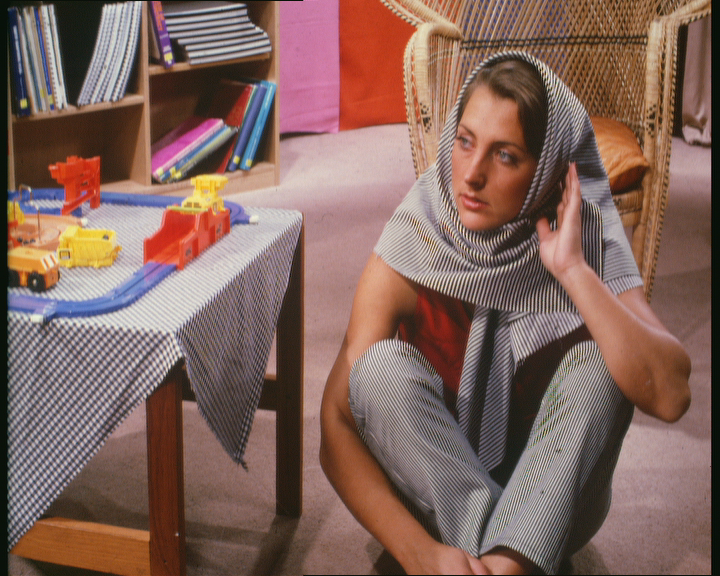

Supplement: S1 Dataset — (ZIP) [file pone.0201463.s007.zip › data/Set14/barbara.bmp]

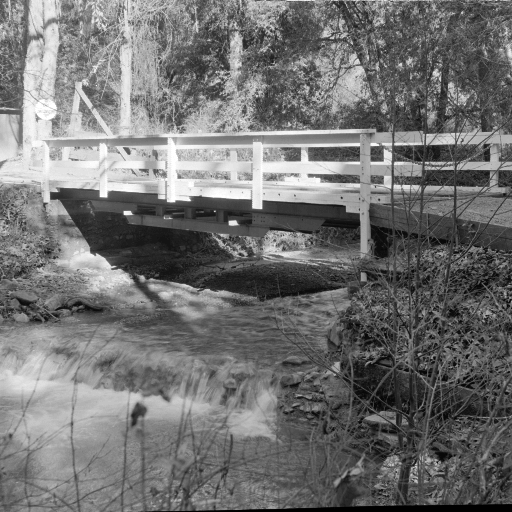

Supplement: S1 Dataset — (ZIP) [file pone.0201463.s007.zip › data/Set14/bridge.bmp]

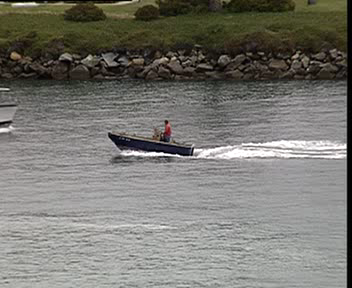

Supplement: S1 Dataset — (ZIP) [file pone.0201463.s007.zip › data/Set14/coastguard.bmp]

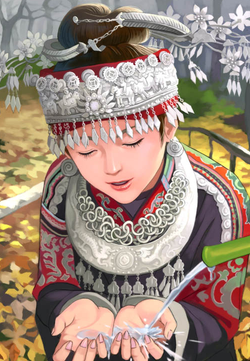

Supplement: S1 Dataset — (ZIP) [file pone.0201463.s007.zip › data/Set14/comic.bmp]

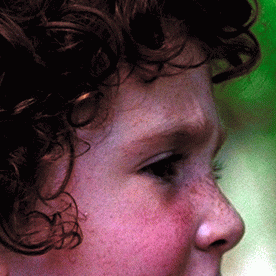

Supplement: S1 Dataset — (ZIP) [file pone.0201463.s007.zip › data/Set14/face.bmp]

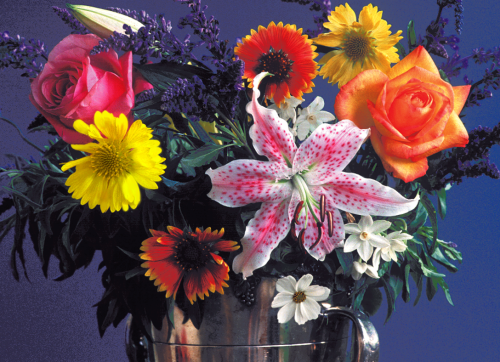

Supplement: S1 Dataset — (ZIP) [file pone.0201463.s007.zip › data/Set14/flowers.bmp]

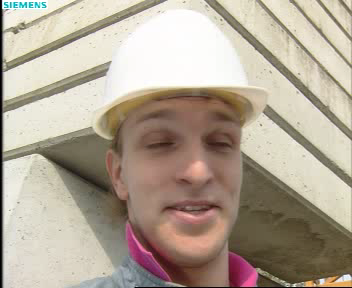

Supplement: S1 Dataset — (ZIP) [file pone.0201463.s007.zip › data/Set14/foreman.bmp]

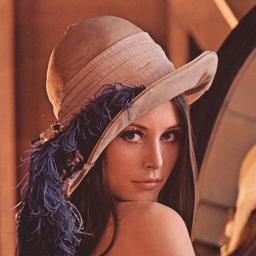

Supplement: S1 Dataset — (ZIP) [file pone.0201463.s007.zip › data/Set14/gnd.bmp]

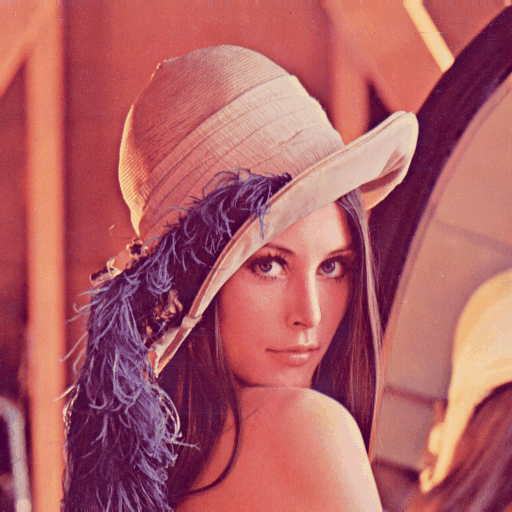

Supplement: S1 Dataset — (ZIP) [file pone.0201463.s007.zip › data/Set14/lenna.bmp]

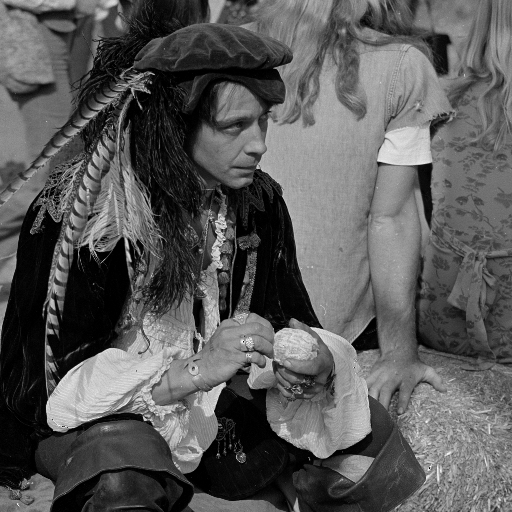

Supplement: S1 Dataset — (ZIP) [file pone.0201463.s007.zip › data/Set14/man.bmp]

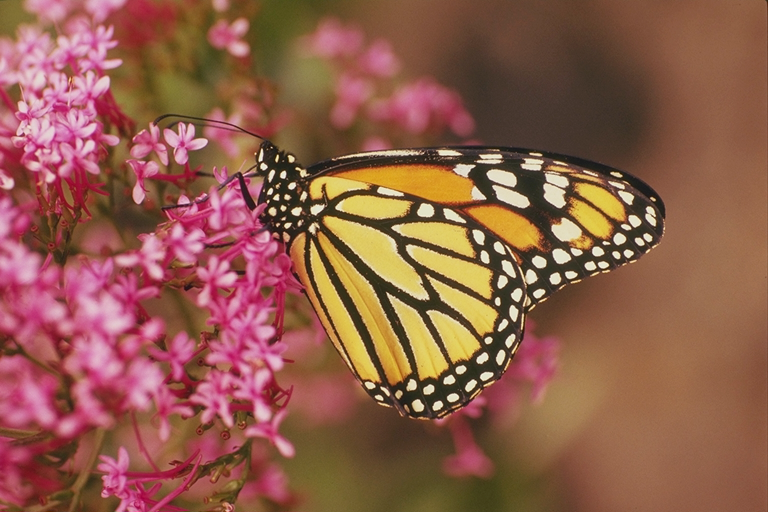

Supplement: S1 Dataset — (ZIP) [file pone.0201463.s007.zip › data/Set14/monarch.bmp]

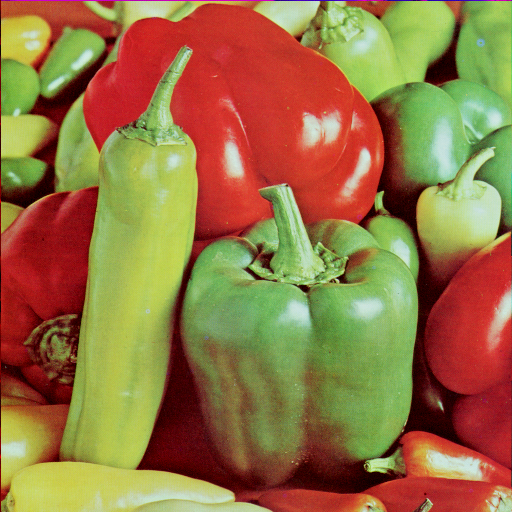

Supplement: S1 Dataset — (ZIP) [file pone.0201463.s007.zip › data/Set14/pepper.bmp]

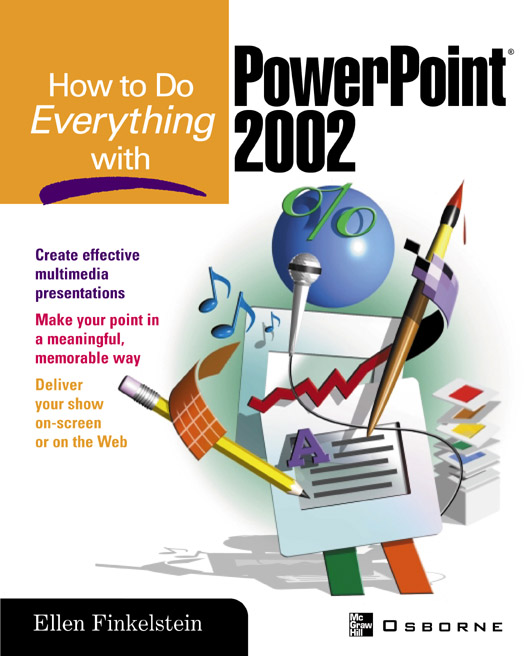

Supplement: S1 Dataset — (ZIP) [file pone.0201463.s007.zip › data/Set14/ppt3.bmp]

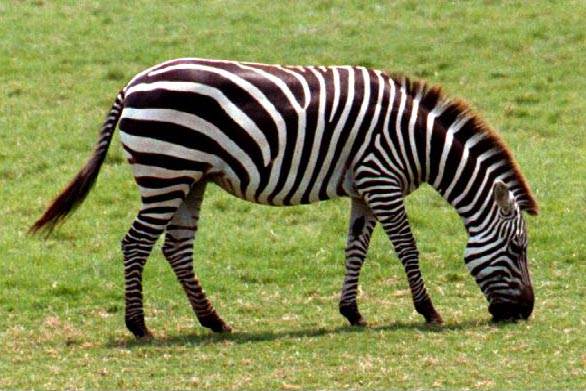

Supplement: S1 Dataset — (ZIP) [file pone.0201463.s007.zip › data/Set14/zebra.bmp]

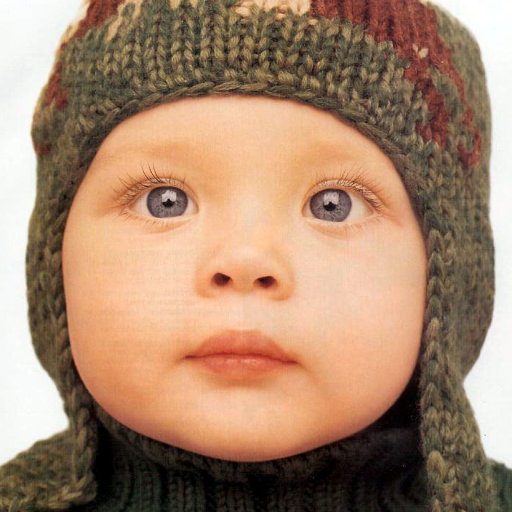

Supplement: S1 Dataset — (ZIP) [file pone.0201463.s007.zip › data/Set5/baby_GT.bmp]

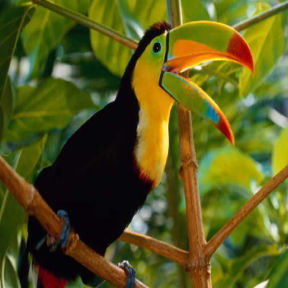

Supplement: S1 Dataset — (ZIP) [file pone.0201463.s007.zip › data/Set5/bird_GT.bmp]

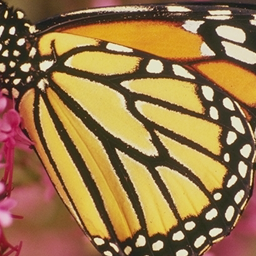

Supplement: S1 Dataset — (ZIP) [file pone.0201463.s007.zip › data/Set5/butterfly_GT.bmp]

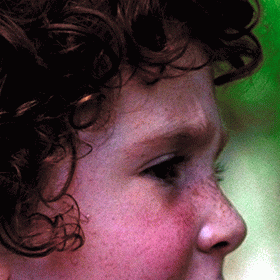

Supplement: S1 Dataset — (ZIP) [file pone.0201463.s007.zip › data/Set5/head_GT.bmp]

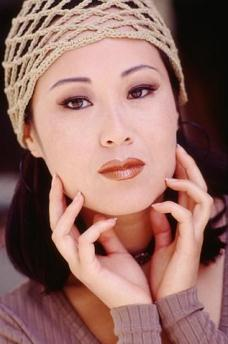

Supplement: S1 Dataset — (ZIP) [file pone.0201463.s007.zip › data/Set5/woman_GT.bmp]
